# Impact of a short-term Mediterranean diet intervention on plasma metabolites: a pilot study

**DOI:** 10.1007/s11306-024-02154-7

**Published:** 2024-07-27

**Authors:** E. Smith, F. Ottosson, U. Ericson, S. Hellstrand, M. Rizzo, K. Sukruang, V. Pizza, M. Orho-Melander, P. M. Nilsson, C. Kennbäck, C. Fernandez, P. Antonini, S. Di Somma, O. Melander

**Affiliations:** 1 Department of Clinical Sciences Malmö, Lund University, Malmö, Sweden; 2 Department of Congenital Disorders, Statens Serum Institut, Copenhagen, Denmark; 3 GREAT Health Sciences, Rome, Italy; 4 Faculty of Medicine Ramathibodi Hospital, Chakri Naruebodindra Medical Institute, Mahidol University, Samut Prakan, Thailand; 5 Institute of Future Studies for Development, Bangkok, Thailand; 6 Department of Emergency and Time Dependent Networks, Neurology Unit, S.Luca Hospital, Vallo Della Lucania, Italy; 7 Department of Emergency and Internal Medicine, Skåne University Hospital, Malmö, Sweden; 8 Department of Medical-Surgery Sciences and Translational Medicine, University of Rome Sapienza, Rome, Italy

**Keywords:** Nutritional metabolomics, Mediterranean diet, Cardiovascular disease, Type 2 diabetes, Nutrition

## Abstract

**Background:**

Dietary habits significantly influence the risks of type 2 diabetes and cardiovascular disease. Through metabolomics, we’ve previously measured plasma metabolites to gauge dietary quality, introducing a healthy dietary metabolic signature (HDMS) linked to a decreased risk of future type 2 diabetes and coronary artery disease.

**Objectives:**

To assess the impact of a 6-day dietary intervention on plasma metabolites and the HDMS.

**Methods:**

Fifty-nine Swedish participants (71% women, mean age 69 years) underwent a 6-day Mediterranean diet (MD) intervention in Italy’s Cilento region. All meals, crafted from local recipes and ingredients, were provided. Metabolite profiling pre- and post-intervention was conducted with a UHPLC-QTOF. Alterations in metabolite levels and the HDMS were examined using paired *T*-test.

**Results:**

The MD intervention notably enhanced the HDMS across participants (mean increase: 1.3 standard deviations (SD), 95% CI 1.1–1.4, p = 6E-25). Out of 109 metabolites, 66 exhibited significant alterations (fdr adjusted p < 0.05). Among the 10 most significant changes, increases were observed in several diet related metabolites such as pipecolate, hippurate, caffeine, homostachydrine, acylcarnitine C11:0, acetylornithine, beta-carotene and 7-methylguanine. The most significant decreases manifested in piperine and 3-methylhistidine.

**Conclusions:**

The HDMS, which is linked to a healthy diet and inversely associated with cardiometabolic disease, was significantly improved by the 6-day Mediterranean diet intervention. Notably, metabolite markers previously shown to be indicative of the intake of vegetables, fruits, grains, and legumes increased, while markers previously associated with red meat consumption decreased. These findings highlight the potential of short-term dietary interventions to induce significant changes in plasma metabolite profiles.

**Supplementary Information:**

The online version contains supplementary material available at 10.1007/s11306-024-02154-7.

## Introduction

Cardiovascular disease (CVD) is the most common non-communicable cause of death globally (Mortality and Causes of Death C., 2015). CVD mortality has fallen over the last 10 years, attributable to enhanced management of risk factor such as hypertension and elevated LDL-cholesterol, reduced smoking prevalence and advancement in treating disease endpoints (Mensah et al., 2017; Townsend et al., 2016). However, this progress is threatened by global increase of obesity and type 2 diabetes (Collaborators et al., 2017; Visseren et al., 2021). Despite being a principal modifiable risk factor of both type 2 diabetes and CVD, dietary habits have shown only marginal improvement (Micha et al., 2017; Miller et al., 2022). Initially identified in the 1960s, the Mediterranean diet’s inverse association with CVD risk has been consistently corroborated in subsequent studies (Estruch et al., 2018; Lorgeril et al., 1999). Current European Society of Cardiology (ESC) guidelines advocate its daily adoption for CVD prevention (Visseren et al., 2021; Widmer et al., 2015).

Dietary assessment methods, such as food frequency questionnaires, dietary recall, and food diaries, are commonly used in clinical practice to identify individuals at high risk of cardiometabolic disease due to unhealthy dietary habits. However, these methods can be time-consuming and may suffer from both systematic and non-systematic errors that decrease the quality of dietary data. Therefore, more efficient and accurate methods are needed (Brennan and Hu, 2019; Noerman and Landberg, 2023; Ulaszewska et al., 2019).

Measurement of a wide range of metabolites (metabolomics) that can be directly influenced by diet has emerged as a promising new tool to completement and improve the accuracy and precision of dietary assessment methods (Scalbert et al., 2014). Additionally, metabolomics can provide valuable information on the intermediate metabolic steps that link dietary intake to the development of chronic diseases, which can help to identify potential targets for intervention and disease prevention. Overall, the use of metabolomics in nutritional research offers a comprehensive and objective approach to studying the relationship between diet, metabolism, and health (Noerman and Landberg, 2023).

We recently identified a metabolic signature associated with a healthy diet and lower risk for coronary artery disease and type 2 diabetes in two large cohorts (Smith et al., 2022). We suggested that metabolomics could be used to identify subgroups who could benefit from dietary interventions and to evaluate adherence to prescribed diets. In this study, we aim to investigate whether a 6-day intervention with a Mediterranean diet can promptly lead to improvements in the metabolic profile previously shown to be associated with healthy eating and protection from cardiometabolic risk (Smith et al., 2022), referred to as the Healthy Dietary Metabolic Signature (HDMS). By examining the extent to which dietary changes can modify the metabolome, we also aim to demonstrate the potential usefulness of metabolomics to assess metabolic alterations after dietary changes.

## Methods

### Intervention description

Our intervention aimed to immerse participants in the authentic experience of consuming a Mediterranean diet, rather than merely providing dietary advice as is common in real-life interventions. To investigate the effects of a 6-day-long exposure to a genuine Mediterranean diet on the HDMS, we invited Swedish volunteers to participate in a leisure trip to the Cilento region of Campania in Southern Italy to focus on the local diet. Throughout the entire intervention, all meals and beverages were provided and prepared onsite using local ingredients.

Sixty Swedish individuals were recruited for the intervention through an advertisement in a Swedish newspaper in collaboration with the travel agency arranging the leisure travel from Sweden to Italy. Interested individuals received further information through mail and were eligible for the study if they provided oral and written informed consent, were at least 18 years old, and were able to pay for the entire Cilento travel (including all meals) themselves. There was no financial compensation for participants. The study was approved by the Swedish Ethical Review Authority (Dnr 2019-06108). The study participants who were included in the study traveled to Cilento region in Italy by air and then by bus, arriving at the study location in the evening before the first day of the intervention.

After an overnight fast starting at 10:00 p.m., baseline sampling was conducted in the morning. This included venous blood sampling, blood pressure measurement, and anthropometric measurements. The samples were promptly frozen to − 20 °C and further to − 80 degrees within 12 h and transported to Malmö, Sweden within 7 days, where they were stored at − 80 degrees before being analyzed. After a 6-day long intervention, identical blood sampling and clinical exams were repeated in the morning after following another overnight fast. The participants then returned to Sweden and no further monitoring was performed. Participants completed questionnaires regarding current medications, smoking status, and prevalent diseases before the intervention. These questionnaires were self-administered.

During the 6-day intervention, participants stayed in a boarding house and all their meals were provided. The meals were prepared by local cooks using local ingredients and were designed to reflect the typical Mediterranean diet of the region. A meal plan can be found in the supplementary material. Except for the breakfasts, which were served as a buffet in the hotel, the various dishes were served directly at the table. Thus, being served locally produced Cilento food for all meals during the entire six days was the main exposure and the change of the HDMS between pre- and post- 6 days intervention was the primary outcome measure. There was no food questionnaire or other measure to control which of the served items, and how much of various food items were ingested. Additionally, there was no measurement of the participants’ dietary intake before the intervention.

### Basic lab analyses

Plasma concentrations of creatinine, total cholesterol, LDL cholesterol, HDL cholesterol, triglycerides, glucose, haemoglobin A_1C_ and urea were assessed in both baseline and post-interventional samples at D’Arena s.r.l., Vallo Della Lucania, Italy, using accredited laboratory methods. The laboratory holds accreditation from the NHS, ensuring that all tests conducted for the study adhere to rigorous standards. This accreditation signifies compliance not only with the essential prerequisites but also with additional self-certified requirements, meticulously validated by both a dedicated regional commission and a supplementary commission evaluating the operational capabilities of the facility. These assessments lead to the assignment of COMs (Certificates of Measurement) which consider the laboratory’s production capacity, including the range of instrumentation and the workload managed by staff across various departments.

### Metabolite measurements

Metabolomics analysis Profiling of plasma metabolites was performed using LC–MS using a UPLC-QTOF-MS System (Agilent Technologies 1290 LC, 6546 MS, Santa Clara, CA, USA) and has been described elsewhere (Ottosson et al., 2016; Smith et al., 2022). Briefly, the fasted plasma samples were extracted and subsequently separated on an Acquity UPLC BEH Amide column (1.7 μm, 2.1 × 100 mm; Waters Corporation, Milford, MA, USA) before being analysed in positive ion mode. The sample run order was randomized and the pre- and post-intervention samples were run in the same batch. Quality control samples were injected every six samples.

We identified metabolites by matching the measured mass-over-charge ratio (m/z) and chromatographic retention times with an in-house metabolite library consisting of 111 metabolites. Annotations followed the Metabolomics Standard Initiative guidelines (Sumner et al., 2007) (supplemental Table 1).

**Table 1:** Clinical characteristics pre- and post-intervention

	Mean Before (SD) or n (%)	Mean After (SD)	fdr-adjusted p
Age, years	69.4 (9.2)	–	NA
Women	42 (71%)	–	NA
Smoking	2 (3%)	–	NA
Type 2 diabetes	5 (8%)	–	NA
Atrial fibrillation	3 (5%)	–	NA
Lipid lowering medication	6 (10%)	–	NA
Anti-hypertensive medication	12 (20%)	–	NA
Height, cm	170 (8.3)	–	NA
Weight, kg	70.2 (10.2)	70.5 (10.1)	0.2
BMI, kg/m^2^	24.4 (2.7)	24.5 (2.7)	0.2
SBP mmHg	131.3 (19.2)	132.7 (20.4)	1
DBP mmHg	86.6 (12.5)	85.7 (12.5)	1
Resting HR	73.5 (12.6)	74.0 (14.7)	1
Urea (mg/dl)	38.6 (8.8)	28.4 (7.2)	< 0.001
Creatinine (µmol/L)	74.1 (13.7)	74.0 (12.3)	1
Cholesterol total (mmol/L)	6.2 (1.1)	6.0 (1.0)	0.08
LDL (mmol/L)	3.5 (1.0)	3.4 (0.8)	1
HDL (mmol/L)	2.0 (0.5)	1.9 (0.5)	0.6
Triglycerides (mmol/L)	2.5 (0.9)	2.3 (2.3)	0.2
Glucose (mmol/L)	5.1 (0.8)	5.3 (5.3)	0.2
HbA1c (mmol/mol)	42.8 (5.3)	41.7 (5.4)	0.4

Means and standard deviations (SD) of clinical characteristics before and after the 6-day intervention. Mean differences were tested using paired t-test adjusted. BMI: Body mass index. SBP: Systolic blood pressure. DBP: Diastolic blood pressure. HR: Heart rate. LDL: Low density lipoprotein. HDL: High density lipoprotein. HbA1C: hemoglobin A1C

In this study, we measured 109 metabolites from an in-house library using Agilent Profinder B.06.00 (Agilent Technologies, Santa Clara, CA, USA) and visual inspection by an experienced user. We normalized the metabolite measurements using standard curves calculated from quality control samples and applied a low-order nonlinear locally estimated smoothing function to correct for injection order (Dunn et al., 2011). Outliers and measurements below the limit of detection were excluded by setting a cut-off of 5 standard deviations. These outliers were imputed to − 5 or + 5 standard deviations. The normalized, scaled data were then mean-centered and unit-variance scaled.

### HDMS

We calculated HDMS according to a previously published partial least square regression model created in the Malmö Offspring Study (Smith et al., 2022). In that study, the HDMS was created as a model using all annotated metabolites in our inhouse library (n = 111) trained to predict a previously reported health-conscious dietary pattern (Ericson et al., 2020).The health-conscious dietary pattern was characterized by high intakes of fruits and berries, nuts and seeds, legumes, other vegetables (nonlegumes), plain yogurt, fresh cheese, tea, animal replacement foods, breakfast cereals, cooked grains such as bulgur, oil-based dressings, fish and fiber-rich bread, and by low intakes of sugar-sweetened beverages, red and processed meat, white bread and fried/deep-fried potatoes (Ericson et al., 2020). The Mediterranean diet intervention similarly emphasized high intakes of fruits, vegetables, whole grains, nuts, seeds, and olive oil, while also promoting low intakes of red meat and processed foods. Both diets align closely in promoting plant-based foods and minimizing processed and red meat consumption.

The resulting HDMS based on the health conscious-dietary pattern in Malmö Offspring study (n = 1538) was shown to associate with a lower incidence of coronary artery disease and type 2 diabetes in two different cohorts (Malmö Diet and Cancer n = 2521 and Malmö Preventive Project n = 1083) (Smith et al., 2022). We applied the “Predict” function in the R package mixOmics to estimate the HDMS pre- and post-interventional values of the participants in the intervention based on the individual metabolite levels (Rohart et al., 2017). Out of the 111 metabolites in the original pattern, valine and aminoisobutyrate were not measurable in the current study and the score was therefore calculated on the remaining 109 metabolite levels. The contribution of valine and aminoisobutyrate to the original pattern were low (Smith et al., 2020).

### Statistical analysis

R (version 4.2.1) was used for all statistical analyses. To evaluate the potential changes in HDMS, individual metabolite values and clinical parameters from baseline to post-intervention, we used paired t-tests and applied false discovery rate (fdr) correction to adjust for multiple testing (Benjamini and Hochberg, 2018).

## Results

A total of 59 individuals completed the intervention. The mean age of the participants was 69 years, 71% were women and their mean BMI was 24.4 (Table 1).

There were no statistically significant changes over the 6 days exposure to the Mediterranean diet in body weight, body mass index (BMI), or systolic and diastolic blood pressure. Out of the routine laboratory tests, urea levels decreased significantly by 10.3 mg/dl (95% CI − 8.6 to − 11.9, p-value = 4E-16) while creatinine, cholesterols, triglycerides, glucose, and haemoglobin A1C did not show significant changes between pre- and post-intervention.

### HDMS improvement in all participants

**Fig. 1:**
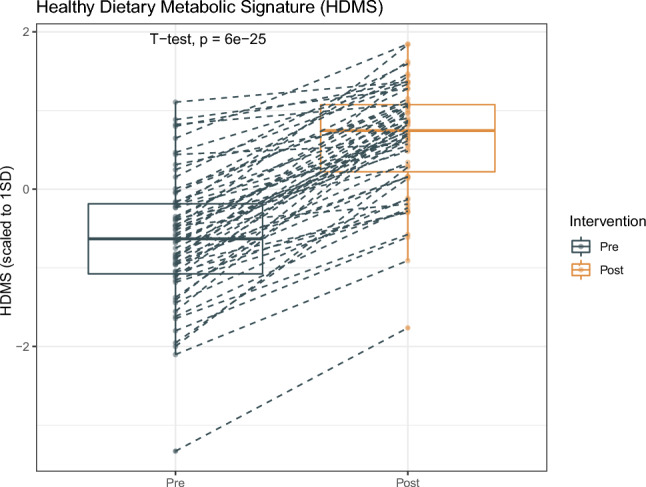
HDMS variations following the 6-day intervention. Connecting lines represent the transition from pre-intervention to post-intervention HDMS values for each participant. The HDMS values are mean centred and scaled in units of 1 standard deviation (SD)

The intervention resulted in an improvement in the HDMS for all participants, and the overall change was statistically significant with a large average effect size (mean SD increase 1.3, 95% CI 1.1*—*1.4, p = 6E-25) (Fig. 1).

Out of the 10 most positively contributing metabolites in the original publication, i.e. metabolites associated with a health-conscious food pattern in the population-based setting (Smith et al., 2022), eight exhibited significantly higher levels post intervention, while one showed significant decrease (supplemental Table 2). Conversely, from the 10 metabolites that most negatively influenced the original model, three (urobilin, acylcarnitine C8:1 and acylcarnitine C10:3) demonstrated significant decrease, and one (creatinine) had a notable increase (supplemental Table 2).

**Table 2:** Most significant metabolite alterations

Metabolite	Mean difference (95% CI)	Fdr adjusted p	Original loading
Pipecolate	1.6 (1.5—1.8)	8E-26	0.11
Piperine	− 1.4 (− 1.5—− 1.2)	1E-21	− 0.04
Hippurate	1.3 (1.1—1.5)	9E-17	0.17
Caffeine	1.2 (1—1.4)	7E-14	0.01
Homostachydrine	1.1 (0.9—1.3)	2E-13	0.21
3-Methyl-histidine	− 1.4 (− 1.7—− 1.1)	1E-12	0.004
C11:0-acylcarnitine	1.1 (0.9—1.4)	9E-12	0.12
Acetylornithine	0.7 (0.5—0.8)	2E-10	0.20
Beta-carotene	0.9 (0.6—1.1)	5E-10	0.36
7-Methylguanine	0.7 (0.5—0.9)	7E-10	− 0.09
C14:1-acylcarnitine	0.9 (0.6—1.1)	1E-09	0.03
Paraxanthine	1.0 (0.7—1.3)	1E-09	0.04

Top 12 metabolite alterations, represented in units of one standard deviation (SD). These changes were determined using a paired T-test and adjusted for false discovery rate (fdr). The ‘original loading’ column corresponds to the loading in the original HDMS (Smith et al., 2022). Comprehensive data on all evaluated metabolites can be found in Supplemental Table 1

### Individual metabolite changes

**Fig. 2:**
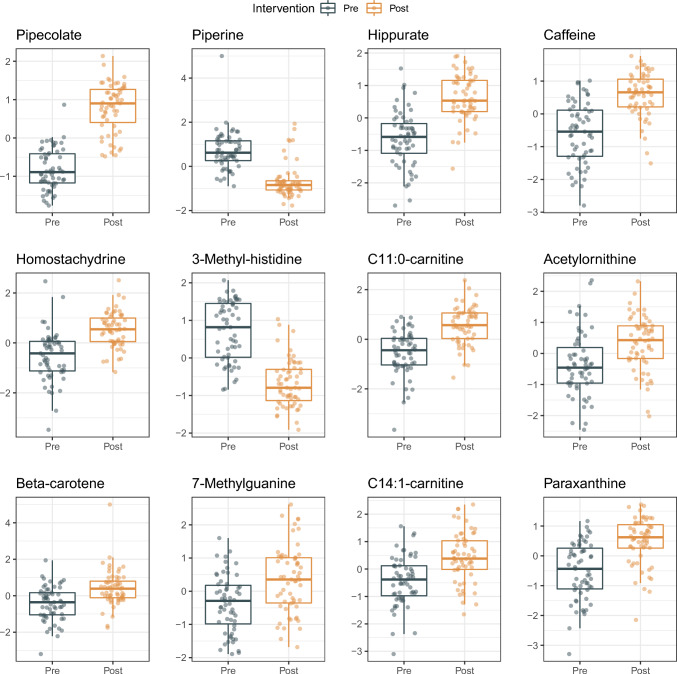
Top 12 most significant metabolite alterations. The chart displays metabolite levels before and after intervention, showcasing individual data points alongside boxplots. On the Y-axis, metabolite levels are mean-centred and scaled in units of one standard deviation (SD)

Out of the 111 measured metabolites in the previous publication (Smith et al., 2022), 109 were measurable in this intervention while only valine and aminoisobutyrate were not. Out of 109 measured metabolites, 66 were significantly altered (p fdr < 0.05) (supplemental Table 1). The most significantly increased levels were seen in pipecolate (1,6 SD increase, 95% CI 1.5—1.8, p = 7E-28), hippurate (1.3 SD, CI 1.1—1.5, p = 2E-18). Other metabolites with significant increases include caffeine, homostachydrine, acylcarnitine C11:0, acetylornithine, beta-carotene, 7-methylguanine, acylcarnitine C14:1 and paraxanthine (Fig. 2 and Table 2). The most significantly decreased levels were seen in piperine (1.4 SD decrease, 95% CI − 1.4—− 1.2, p = 2E-18) and 3-methylhistidine (1.4 decrease, 95% CI − 1.7—− 1.1, p = 7E-10) (Fig. 2 and Table 2). All metabolite changes are available in supplementary Table 1.

In the list of the 12 notable changes highlighted earlier, five metabolites—pipecolate, hippurate, homostachydrine, acylcarnitine C11:0, and beta-carotene had a high impact on the original HDMS, with loadings either exceeding 0.10 or falling below − 0.10 (as detailed in Table 2). Each of these five metabolites showed a positive contribution to the original HDMS (with a loading greater than 0.10) and experienced an increase following the intervention.

## Discussion

### Key findings

In our 6-day intervention, Swedish participants travelled to Cilento, Italy, and consumed local food, resulting in a marked improvement of the HDMS. The HDMS has been previously associated with healthy eating habits and protection from both type 2 diabetes and coronary artery disease (Smith et al., 2022). Moreover, the intervention had a significant impact individually on the majority of the measured metabolites. We find it confidence-inspiring, given the nature of the intervention, that the most notable changes were observed in metabolites associated with dietary intake, many of which derived from dietary items themselves.

### Clinical implication of HDMS improvement

Our findings revealed a notable enhancement in the health-conscious eating correlated HDMS following a 6-day Mediterranean diet intervention. The HDMS, previously established and validated through two extensive population-based studies in Malmö, Sweden, inversely correlates with the incidence of coronary artery disease and type 2 diabetes, indicating that higher HDMS values are associated with reduced disease risk (Smith et al., 2022). While previous studies have demonstrated that a Mediterranean diet can alter the metabolic profile (Meslier et al., 2020; Sobiecki et al., 2023), our study is the first to show that such a short-term intervention can lead to measurable improvements in the HDMS. This underscores the potential effectiveness of even brief dietary changes in positively influencing metabolic health markers and highlights the utility of the HDMS as a tool for assessing the impact of dietary interventions although further research is required for validation. It is important to acknowledge that while the metabolites analyzed in this study have been associated with low cardiovascular risk in different populations, the same associations may not necessarily apply to this specific population. Similarly, associations with food intake observed in other populations might not be directly transferable to the current study participants.

The absence of baseline dietary data limits the precise evaluation of the intervention’s individual impact. Further, the study’s volunteer group, being more likely to have an inherent interest in dietary health, may not be entirely representative of the general population, potentially affecting the generalizability of our results.

Nevertheless, the positive changes in the metabolome across all participants highlight the potential of dietary modifications to influence metabolic profiles. This finding suggests the feasibility of using HDMS in long-term trials to monitor dietary adherence and improve outcomes, especially considering its rapid responsiveness compared to traditional clinical markers like body weight and blood pressure.

### Discussion of top metabolites and previous research

Significant changes in metabolites indicative of dietary intake were observed. Pipecolate, a marker for dry bean consumption (Perera et al., 2015) showed a marked increase, reflecting the bean-rich meals during the intervention. Piperine, linked to processed meat intake, decreased significantly suggesting lower than habitual intake of of processed meat during the intervention (Wedekind et al., 2021).

Hippurate has been linked to the consumption of dietary items rich in polyphenolic compounds such as fruit, berries, and coffee (Hanhineva et al., 2015; Rafiq et al., 2021) and increased with the intervention. Homostachydrine, previously associated with whole grain intake (Playdon et al., 2017) also increased after the intervention, reflecting the numerous meals containing whole grain flours. Conversely, 3-methyl-histidine, that correlates with poultry and meat consumption, declined, suggesting reduced intake of these foods (Kochlik et al., 2018; Rafiq et al., 2021).

Increases in acetylornithine and beta carotene, linked to fruit and vegetable consumption, were observed (Hellstrand et al., 2021; Playdon et al., 2017; Rafiq et al., 2021). However, ergothioneine levels, associated with vegetable consumption and reduced mortality and CVD were unchanged (Smith et al., 2020). Ergothioneine supplementation reaches its maximum effect on plasma levels after more than seven days, which might explain the absence of significant change in this metabolite (Cheah et al., 2017).

### Components of the metabolic signature and their changes

The improvement in the HDMS during the intervention was primarily due changes in metabolites with previously shown positive associations to the HDMS (Smith et al., 2022). Of the top 10 previously positively associated metabolites in the original model, 8 showed significant enhancements, while only 3 of the top 10 negative metabolites decreased. The positively associated metabolites in the original model are typically linked with specific dietary items whereas the negative metabolites are more linked with adverse cardiometabolic health (Smith et al., 2022). This suggests that a longer intervention, might be required to significantly impact metabolites related to cardiometabolic health, as the short duration of this study, as expected, did not markedly affect clinical risk factors such as BMI or systolic blood pressure.

### Shift in clinical parameters

In clinical parameters, a significant decrease in urea, a byproduct of dietary protein breakdown inversely related to eGFR, was noted. This reduction likely reflects a lower protein intake during the intervention compared to the participants’ usual diet, given urea’s correlation with protein consumption (Young et al., 2000).

### Limitations

Our study faces several limitations. Firstly, metabolite measurements were conducted in relative, not absolute, concentration, limiting the reproducibility. Future research could benefit from a focused panel of biomarkers that could be quantified using standardized methods for more accurate results.

Secondly, the lack of baseline dietary data hinders the precise assessment of the Mediterranean diet’s impact. The participant group, a small cohort of Swedish volunteers may not represent the broader population, given their distinct dietary preferences, health interest compared to the general population. This specificity, together with the assumed Nordic dietary background of the participants, suggests that the results may not fully translate to individuals with different cultural or dietary backgrounds, or those with specific health conditions. Due to the limited sample size and short length of the intervention, our study should be considered as a pilot study that encourages replication.

Another limitation of our study is the lack of tight control over the participants’ dietary intake during the intervention. While the meals provided were based on the Mediterranean diet, we did not monitor the quantity of each meal consumed by the participants. This lack of control could introduce variability in the results and may affect the reproducibility of the study. However, this approach mirrors real-life settings more closely than controlled feeding studies, offering insights into adherence to dietary recommendations in everyday life. In future research, it might be worthwhile to explore the effects of dietary advice on the HDMS. This would provide valuable insights into how well individuals adhere to a prescribed diet when following recommendations, as opposed to consuming meals prepared for them, which would more closely resemble real-life interventions and better inform public health strategies.

## Conclusion

In summary, our study demonstrates that the HDMS, which previously has been linked to a healthy diet and inversely associated with cardiometabolic disease, was significantly improved by the six-day Mediterranean diet intervention. Notably, metabolite markers previously shown to be indicative of the intake of vegetables, fruits, grains, and legumes increased, while markers previously associated with red meat consumption decreased. These findings underscore the potential impact of short-term dietary interventions on plasma metabolite profiles, suggesting that metabolomic analysis can be a valuable tool for assessing dietary intervention efficacy. Further investigation is necessary to validate these results and to assess the long-term effects on both the metabolome and cardiometabolic health.

### Supplementary Information

Below is the link to the electronic supplementary material.

Supplementary file1 (DOCX 49 KB)

## Data Availability

The data supporting the findings of this study are available from the corresponding author upon reasonable request. Requests for access to these data should be directed to the corresponding author, who will consider such inquiries in accordance with the ethical guidelines and legal regulations governing research data confidentiality and participant privacy.

## Abbreviations

CVD: Cardiovascular Disease
HDMS: Healthy dietary metabolic signature
SD: Standard deviation

## References

[CR1] Benjamini, Y., & Hochberg, Y. (2018). Controlling the false discovery rate: A practical and powerful approach to multiple testing. *Journal of the Royal Statistical Society: Series B (methodological),**57*(1), 289–300. 10.1111/j.2517-6161.1995.tb02031.x 10.1111/j.2517-6161.1995.tb02031.x

[CR2] Brennan, L., & Hu, F. B. (2019). Metabolomics-based dietary biomarkers in nutritional epidemiology-current status and future opportunities. *Molecular Nutrition and Food Research,**63*(1), e1701064. 10.1002/mnfr.201701064 29688616 10.1002/mnfr.201701064

[CR3] Cheah, I. K., Tang, R. M., Yew, T. S., Lim, K. H., & Halliwell, B. (2017). Administration of pure ergothioneine to healthy human subjects: uptake, metabolism, and effects on biomarkers of oxidative damage and inflammation. *Antioxidants and Redox Signaling,**26*(5), 193–206. 10.1089/ars.2016.6778 27488221 10.1089/ars.2016.6778

[CR4] Collaborators, G. B. D. O., Afshin, A., Forouzanfar, M. H., Reitsma, M. B., Sur, P., Estep, K., et al. (2017). Health effects of overweight and obesity in 195 countries over 25 years. *The New England Journal of Medicine,**377*(1), 13–27. 10.1056/NEJMoa1614362 28604169 10.1056/NEJMoa1614362 PMC5477817

[CR5] de Lorgeril, M., Salen, P., Martin, J. L., Monjaud, I., Delaye, J., & Mamelle, N. (1999). Mediterranean diet, traditional risk factors, and the rate of cardiovascular complications after myocardial infarction: Final report of the Lyon diet heart study. *Circulation,**99*(6), 779–785. 10.1161/01.cir.99.6.779 9989963 10.1161/01.cir.99.6.779

[CR6] Dunn, W. B., Broadhurst, D., Begley, P., Zelena, E., Francis-McIntyre, S., Anderson, N., et al. (2011). Procedures for large-scale metabolic profiling of serum and plasma using gas chromatography and liquid chromatography coupled to mass spectrometry. *Nature Protocols,**6*(7), 1060–1083. 10.1038/nprot.2011.335 21720319 10.1038/nprot.2011.335

[CR7] Ericson, U., Brunkwall, L., Hellstrand, S., Nilsson, P. M., & Orho-Melander, M. (2020). A health-conscious food pattern is associated with prediabetes and gut microbiota in the malmo offspring study. *Journal of Nutrition,**150*(4), 861–872. 10.1093/jn/nxz293 31851320 10.1093/jn/nxz293 PMC7138670

[CR8] Estruch, R., Ros, E., Salas-Salvado, J., Covas, M. I., Corella, D., Aros, F., et al. (2018). Primary prevention of cardiovascular disease with a Mediterranean diet supplemented with extra-virgin olive oil or nuts. *The New England Journal of Medicine,**378*(25), e34. 10.1056/NEJMoa1800389 29897866 10.1056/NEJMoa1800389

[CR9] Hanhineva, K., Lankinen, M. A., Pedret, A., Schwab, U., Kolehmainen, M., Paananen, J., et al. (2015). Nontargeted metabolite profiling discriminates diet-specific biomarkers for consumption of whole grains, fatty fish, and bilberries in a randomized controlled trial. *Journal of Nutrition,**145*(1), 7–17. 10.3945/jn.114.196840 25527657 10.3945/jn.114.196840

[CR10] Hellstrand, S., Ottosson, F., Smith, E., Brunkwall, L., Ramne, S., Sonestedt, E., et al. (2021). Dietary data in the Malmo offspring study-reproducibility, method comparison and validation against objective biomarkers. *Nutrients,**13*(5), 1579. 10.3390/nu13051579 34065043 10.3390/nu13051579 PMC8150333

[CR11] Kochlik, B., Gerbracht, C., Grune, T., & Weber, D. (2018). The Influence of dietary habits and meat consumption on plasma 3-methylhistidine—A potential marker for muscle protein turnover. *Molecular Nutrition and Food Research,**62*(9), e1701062. 10.1002/mnfr.201701062 29573154 10.1002/mnfr.201701062 PMC5969234

[CR12] Mensah, G. A., Wei, G. S., Sorlie, P. D., Fine, L. J., Rosenberg, Y., Kaufmann, P. G., et al. (2017). Decline in cardiovascular mortality: Possible causes and implications. *Circulation Research,**120*(2), 366–380. 10.1161/CIRCRESAHA.116.309115 28104770 10.1161/CIRCRESAHA.116.309115 PMC5268076

[CR13] Meslier, V., Laiola, M., Roager, H. M., De Filippis, F., Roume, H., Quinquis, B., et al. (2020). Mediterranean diet intervention in overweight and obese subjects lowers plasma cholesterol and causes changes in the gut microbiome and metabolome independently of energy intake. *Gut,**69*(7), 1258–1268. 10.1136/gutjnl-2019-320438 32075887 10.1136/gutjnl-2019-320438 PMC7306983

[CR14] Micha, R., Penalvo, J. L., Cudhea, F., Imamura, F., Rehm, C. D., & Mozaffarian, D. (2017). Association between dietary factors and mortality from heart disease, stroke, and type 2 diabetes in the United States. *JAMA,**317*(9), 912–924. 10.1001/jama.2017.0947 28267855 10.1001/jama.2017.0947 PMC5852674

[CR15] Miller, V., Webb, P., Cudhea, F., Shi, P., Zhang, J., Reedy, J., et al. (2022). Global dietary quality in 185 countries from 1990 to 2018 show wide differences by nation, age, education, and urbanicity. *Nat Food.,**3*(9), 694–702. 10.1038/s43016-022-00594-9 37118151 10.1038/s43016-022-00594-9 PMC10277807

[CR16] Mortality, G. B. D., & Causes of Death, C. (2015). Global, regional, and national life expectancy, all-cause mortality, and cause-specific mortality for 249 causes of death, 1980–2015: a systematic analysis for the global burden of disease study 2015. *The Lancet,**388*(10053), 1459–1544. 10.1016/S0140-6736(16)31012-1 10.1016/S0140-6736(16)31012-1 PMC5388903 27733281

[CR17] Noerman, S., & Landberg, R. (2023). Blood metabolite profiles linking dietary patterns with health-Toward precision nutrition. *Journal of Internal Medicine,**293*(4), 408–432. 10.1111/joim.13596 36484466 10.1111/joim.13596

[CR18] Ottosson, F., Ericson, U., Almgren, P., Nilsson, J., Magnusson, M., Fernandez, C., & Melander, O. (2016). Postprandial levels of branch chained and aromatic amino acids associate with fasting Glycaemia. *Journal of Amino Acids,**2016*, 8576730. 10.1155/2016/8576730 27274867 10.1155/2016/8576730 PMC4871975

[CR19] Perera, T., Young, M. R., Zhang, Z., Murphy, G., Colburn, N. H., Lanza, E., Hartman, T. J., Cross, A. J., & Bobe, G. (2015). Identification and monitoring of metabolite markers of dry bean consumption in parallel human and mouse studies. *Molecular Nutrition and Food Research,**59*(4), 795–806. 10.1002/mnfr.201400847 25641932 10.1002/mnfr.201400847 PMC4417744

[CR20] Playdon, M. C., Moore, S. C., Derkach, A., Reedy, J., Subar, A. F., Sampson, J. N., et al. (2017). Identifying biomarkers of dietary patterns by using metabolomics. *The American Journal of Clinical Nutrition.,**105*(2), 450–465. 10.3945/ajcn.116.144501 28031192 10.3945/ajcn.116.144501 PMC5267308

[CR21] Rafiq, T., Azab, S. M., Teo, K. K., Thabane, L., Anand, S. S., Morrison, K. M., de Souza, R. J., & Britz-McKibbin, P. (2021). Nutritional metabolomics and the classification of dietary biomarker candidates: A critical review. *Advances in Nutrition,**12*(6), 2333–2357. 10.1093/advances/nmab054 34015815 10.1093/advances/nmab054 PMC8634495

[CR22] Rohart, F., Gautier, B., Singh, A., & Le Cao, K. A. (2017). mixOmics: An R package for ‘omics feature selection and multiple data integration. *PLoS Computational Biology,**13*(11), e1005752. 10.1371/journal.pcbi.1005752 29099853 10.1371/journal.pcbi.1005752 PMC5687754

[CR23] Scalbert, A., Brennan, L., Manach, C., Andres-Lacueva, C., Dragsted, L. O., Draper, J., Rappaport, S. M., van der Hooft, J. J., & Wishart, D. S. (2014). The food metabolome: A window over dietary exposure. *The American Journal of Clinical Nutrition.,**99*(6), 1286–1308. 10.3945/ajcn.113.076133 24760973 10.3945/ajcn.113.076133

[CR24] Smith, E., Ottosson, F., Hellstrand, S., Ericson, U., Orho-Melander, M., Fernandez, C., & Melander, O. (2020). Ergothioneine is associated with reduced mortality and decreased risk of cardiovascular disease. *Heart,**106*(9), 691–697. 10.1136/heartjnl-2019-315485 31672783 10.1136/heartjnl-2019-315485 PMC7229907

[CR25] Smith, E., Ericson, U., Hellstrand, S., Orho-Melander, M., Nilsson, P. M., Fernandez, C., Melander, O., & Ottosson, F. (2022). A healthy dietary metabolic signature is associated with a lower risk for type 2 diabetes and coronary artery disease. *BMC Medicine,**20*(1), 122. 10.1186/s12916-022-02326-z 35443726 10.1186/s12916-022-02326-z PMC9022292

[CR26] Sobiecki, J. G., Imamura, F., Davis, C. R., Sharp, S. J., Koulman, A., Hodgson, J. M., et al. (2023). A nutritional biomarker score of the Mediterranean diet and incident type 2 diabetes: Integrated analysis of data from the MedLey randomised controlled trial and the EPIC-InterAct case-cohort study. *PLoS Medicine.,**20*(4), e1004221. 10.1371/journal.pmed.1004221 37104291 10.1371/journal.pmed.1004221 PMC10138823

[CR27] Sumner, L. W., Urbanczyk-Wochniak, E., & Broeckling, C. D. (2007). Metabolomics data analysis, visualization, and integration. *Methods in Molecular Biology (clifton, NJ),**406*, 409–436. 10.1007/978-1-59745-535-0_20 10.1007/978-1-59745-535-0_20 18287705

[CR28] Townsend, N., Wilson, L., Bhatnagar, P., Wickramasinghe, K., Rayner, M., & Nichols, M. (2016). Cardiovascular disease in Europe: Epidemiological update 2016. *European Heart Journal,**37*(42), 3232–3245. 10.1093/eurheartj/ehw334 27523477 10.1093/eurheartj/ehw334 PMC13480964

[CR29] Ulaszewska, M. M., Weinert, C. H., Trimigno, A., Portmann, R., Andres Lacueva, C., Badertscher, R., et al. (2019). Nutrimetabolomics: An integrative action for metabolomic analyses in human nutritional studies. *Molecular Nutrition and Food Research,**63*(1), e1800384. 10.1002/mnfr.201800384 30176196 10.1002/mnfr.201800384

[CR30] Visseren, F. L. J., Mach, F., Smulders, Y. M., Carballo, D., Koskinas, K. C., Back, M., et al. (2021). 2021 ESC Guidelines on cardiovascular disease prevention in clinical practice. *European Heart Journal,**42*(34), 3227–3337. 10.1093/eurheartj/ehab484 34458905 10.1093/eurheartj/ehab484

[CR31] Wedekind, R., Keski-Rahkonen, P., Robinot, N., Viallon, V., Rothwell, J. A., Boutron-Ruault, M. C., et al. (2021). Pepper Alkaloids and processed meat intake: results from a randomized trial and the European prospective investigation into cancer and nutrition (EPIC) cohort. *Molecular Nutrition and Food Research,**65*(7), e2001141. 10.1002/mnfr.202001141 33592132 10.1002/mnfr.202001141

[CR32] Widmer, R. J., Flammer, A. J., Lerman, L. O., & Lerman, A. (2015). The Mediterranean diet, its components, and cardiovascular disease. *The American Journal of Medicine,**128*(3), 229–238. 10.1016/j.amjmed.2014.10.014 25447615 10.1016/j.amjmed.2014.10.014 PMC4339461

[CR33] Young, V. R., El-Khoury, A. E., Raguso, C. A., Forslund, A. H., & Hambraeus, L. (2000). Rates of urea production and hydrolysis and leucine oxidation change linearly over widely varying protein intakes in healthy adults. *Journal of Nutrition,**130*(4), 761–766. 10.1093/jn/130.4.761 10736327 10.1093/jn/130.4.761

