# Supplementary material for: Impact of a short-term Mediterranean diet intervention on plasma metabolites: a pilot study
Source: Metabolomics. 2024 Jul 27;20(4):82. doi: 10.1007/s11306-024-02154-7 (PMC11283393; doi:10.1007/s11306-024-02154-7)
Supplement: Supplementary file 1 — Supplementary file1 (DOCX 49 KB) [file 11306_2024_2154_MOESM1_ESM.docx]

# Impact of a Short-Term Mediterranean Diet Intervention on Plasma Metabolites: A Pilot Study – supplementary material

# Description of dietary intake

The breakfast buffet boasted a rich variety, featuring eggs, oats, spelt, almonds, walnuts, goat milk and cheese, whole grain toast with tomato and olive oil, and roasted aubergine, among others. Dinners were composed of multiple courses, from appetizers to desserts, each meticulously crafted to highlight the distinct flavors of the Cilento region. Lunches, similarly, blended a mix of appetizers and main courses, echoing the dining experience of the dinners.

**Breakfast Buffet**

- Eggs
- Oats
- Spelt
- Almonds
- Walnuts
- Whole grain rusks with jam or chestnut honey
- Goat milk
- Goat cheese (cacioricotta)
- Whole grain toast with tomato and olive oil
- Roasted aubergine

**During the day (on average):**

- 1 glass of wine for lunch
- 1 glass of wine for dinner
- 30 grams of oil for lunch
- 20 grams of oil for dinner

**DAY 1**

LUNCH

- Cilento appetizer: sauteed escarole, au gratin peppers, baked onion, spelt with carrots, topped with slices of aubergine, courgette and pumpkin
- Cilento pizza
- ancient grain fusilli with courgettes
- mixed salad with carrots, onion and olives
- dried and fresh figs

DINNER

- mixed vegetable appetizer: green beans, roasted eggplant, peppers, potatoes with aromatic herbs, pumpkin
- anchovies with lemon
- fried anchovies
- mixed salad with tomatoes
- focaccia
- black grapes and melon

**DAY 2**

LUNCH

- mixed cold cuts: pork sausage, soppressata, capocollo, pancetta with grilled courgettes and aubergines
- cavatelli with tomato and basil
- aubergines stuffed with goat cheese (cacioricotta)
- white grapes and fresh figs
- Sweet with naspro

DINNER

- Appetizer: Aubergines and courgettes in oil, goat's milk ricotta, buffalo morsel, caciocavallo
- Bean and pumpkin soup
- Mixed salad: Lettuce and tomatoes
- Fruit: Fresh figs, white grapes, prickly pear

**DAY 3**

LUNCH

- Bruschetta with herbs: Toasted bread with rocket, tomatoes, goat cacioricotta, walnuts
- Fried herbs: Basil, sage, courgette flowers
- Lagane and chickpeas: Homemade pasta, chickpeas, rosemary
- Mixed salad: Spelled, salad, purslane, tomatoes, onion, raisins
- Onion susciello (eggs with onion)
- Fruit: Fresh figs, dried figs with syrup

DINNER

- Cilentana anchovies
- Cod au gratin
- Sauteed escarole

**DAY 4**

LUNCH

- Bruschetta with tomato
- Pasta and beans
- Maracucciata: Legume in cream
- Leaves and potatoes: Green leafy vegetables, potatoes, chilli
- Ciambotta: Mixed aubergines, peppers and courgettes
- Mixed salad: Tomatoes, onion
- Fruit: Figs, grapes, prickly pears
- Cilento cake: Sponge cake with cream

DINNER

- Ricotta with red mulberry jam and mint
- Mortella mozzarella
- Crouton with black cabbage
- Crouton with beans
- Parmigiana
- Escarole with raisins
- Salt water with tomato and tuna
- Dried figs

**DAY 5**

LUNCH

- Bruschetta with cherry tomatoes, onion, pepper and celery
- Chestnut fusilli with goat meat sauce
- Beans with chestnuts
- Mixed salad: salad, onion, tomatoes- goat ricotta with jam and honey
- Shepherdesses: fried dough filled with chocolate and chestnuts, covered with honey

DINNER

- Stuffed anchovies
- Marinated anchovies with mint
- Anchovies with breadcrumbs
- Tagliolini with anchovies and fennel
- Mixed salad: tomatoes, carrots and lettuce, onion
- Prickly pears, fresh figs

**DAY 6**

LUNCH

- Vegetable appetizer: mushrooms, grilled peppers, grilled zucchini, grilled pumpkin, escarole and beans, soup and potatoes, baked eggplant
- Ciccimmaretati (legume and grain soup)
- Courgette flower and courgette noodles
- Fig and lemon tart

DINNER

- Long skullcap
- Grilled aubergines and courgettes
- Soup with potatoes
- Cavatelli with chickpeas
- White and black grapes
- Fresh figs

# Supplemental table 1: Measured metabolites with reported changes

| Metabolite | SD change (95% confidence interval) | Fdr-adjusted p | HMDBID | Annotation level | loading |
| --- | --- | --- | --- | --- | --- |
| Pipecolate | 1.61 (1.45 - 1.77) | 8.5E-26 | HMDB00716 | 1 | 0.11 |
| Piperine | -1.38 (-1.55 - -1.21) | 1.0E-21 | HMDB29377 | 1 | -0.04 |
| Hippurate | 1.26 (1.07 - 1.46) | 8.8E-17 | HMDB00714 | 1 | 0.17 |
| Caffeine | 1.19 (0.97 - 1.42) | 7.0E-14 | HMDB01847 | 1 | 0.01 |
| Homostachydrine | 1.06 (0.85 - 1.26) | 1.8E-13 | HMDB33433 | 1 | 0.21 |
| 3-Methylhistidine | -1.39 (-1.68 - -1.11) | 1.5E-12 | HMDB00479 | 1 | 0.004 |
| Acylcarnitine C11:0 | 1.12 (0.88 - 1.36) | 8.9E-12 | HMDB13321 | 2 | 0.12 |
| Acetylornithine | 0.68 (0.52 - 0.84) | 2.0E-10 | HMDB03357 | 1 | 0.20 |
| Beta-carotene | 0.85 (0.64 - 1.06) | 5.5E-10 | HMDB00561 | 1 | 0.36 |
| 7-Methylguanine | 0.72 (0.54 - 0.9) | 7.4E-10 | HMDB00897 | 1 | -0.09 |
| Acylcarnitine C14:1 | 0.87 (0.65 - 1.09) | 1.2E-09 | HMDB0240588 | 2 | 0.03 |
| Paraxanthine | 1.00 (0.74 - 1.26) | 1.3E-09 | HMDB01860 | 1 | 0.04 |
| Acylcarnitine C8:0-OH | 0.80 (0.59 - 1.00) | 2.1E-09 | NA | 2 | 0.04 |
| Creatine | -0.68 (-0.86 - -0.5) | 3.5E-09 | HMDB00064 | 1 | -0.001 |
| Trigonelline | 0.88 (0.65 - 1.12) | 3.9E-09 | HMDB00875 | 1 | 0.11 |
| Acylcarnitine C13:0 | 0.81 (0.59 - 1.04) | 9.9E-09 | HMDB0241308 | 2 | 0.21 |
| Acylcarnitine C12:1 | 0.78 (0.55 - 1.01) | 3.7E-08 | HMDB13326 | 2 | 0.05 |
| Acylcarnitine C10:0 | 0.72 (0.51 - 0.93) | 4.1E-08 | HMDB00651 | 2 | 0.06 |
| Homocitrulline | 0.81 (0.56 - 1.05) | 7.7E-08 | HMDB00679 | 1 | 0.06 |
| Acylcarnitine C10:1 | 0.73 (0.5 - 0.95) | 1.2E-07 | HMDB13205 | 2 | 0.09 |
| Acylcarnitine C12:0 | 0.77 (0.53 - 1.02) | 1.8E-07 | HMDB02250 | 2 | 0.07 |
| Acylcarnitine C10:2 | 0.71 (0.49 - 0.94) | 2.1E-07 | NA | 2 | -0.003 |
| Acylcarnitine C14:2 | 0.78 (0.53 - 1.03) | 2.8E-07 | HMDB13331 | 2 | 0.09 |
| Acylcarnitine C10:3 | -0.67 (-0.90 - -0.44) | 1.4E-06 | NA | 2 | -0.12 |
| Trimethyllysine | -0.77 (-1.05 - -0.48) | 5.9E-06 | HMDB01325 | 1 | -0.10 |
| Acylcarnitine C10:0-OH | 0.61 (0.38 - 0.85) | 1.2E-05 | NA | 2 | 0.05 |
| Acylcarnitine C18:2 | 0.71 (0.44 - 0.98) | 1.2E-05 | NA | 2 | -0.003 |
| Carnitine | -0.62 (-0.85 - -0.38) | 1.3E-05 | HMDB00062 | 1 | -0.10 |
| Acylcarnitine C12:2 | 0.67 (0.41 - 0.93) | 1.3E-05 | NA | 2 | 0.07 |
| Acylcarnitine C2:0 | 0.58 (0.34 - 0.83) | 5.5E-05 | HMDB00201 | 1 | 0.02 |
| Acylcarnitine C8:1 | -0.57 (-0.82 - -0.33) | 7.0E-05 | NA | 2 | -0.12 |
| Acylcarnitine C16:1 | 0.59 (0.34 - 0.85) | 8.5E-05 | HMDB13207 | 2 | 0.04 |
| Acylcarnitine C4:0-OH | 0.47 (0.27 - 0.68) | 8.5E-05 | HMDB13127 | 2 | 0.24 |
| Acisoga | 0.5 (0.28 - 0.72) | 9.4E-05 | HMDB61384 | 1 | 0.05 |
| 1-Methyladenosine | 0.62 (0.35 - 0.89) | 9.5E-05 | HMDB03331 | 1 | 0.02 |
| Acylcarnitine C13:1 | 0.54 (0.29 - 0.79) | 1.7E-04 | NA | 2 | 0.13 |
| Acetylarginine | -0.51 (-0.76 - -0.27) | 3.0E-04 | HMDB04620 | 1 | -0.02 |
| Symmetric dimethylarginine | 0.59 (0.3 - 0.88) | 4.0E-04 | HMDB03334 | 1 | 0.003 |
| Proline betaine | 0.48 (0.23 - 0.72) | 6.9E-04 | HMDB04827 | 1 | 0.15 |
| Phenylacetylglutamine | -0.39 (-0.58 - -0.19) | 6.9E-04 | HMDB06344 | 1 | 0.13 |
| Alanine | -0.51 (-0.77 - -0.24) | 8.8E-04 | HMDB00161 | 1 | -0.02 |
| Acylcarnitine C4:0 | -0.43 (-0.66 - -0.2) | 1.0E-03 | HMDB02013 | 1 | 0.01 |
| Asymmetric dimethylarginine | 0.56 (0.26 - 0.86) | 1.2E-03 | HMDB01539 | 1 | 0.01 |
| Cystine | 0.40 (0.18 - 0.61) | 1.3E-03 | HMDB00192 | 1 | -0.03 |
| Histidine | 0.55 (0.25 - 0.85) | 1.6E-03 | HMDB00177 | 1 | -0.02 |
| N2.N2.Dimethylguanosine | 0.43 (0.19 - 0.67) | 1.9E-03 | HMDB04824 | 1 | -0.08 |
| Acetylcarnosine | 0.34 (0.15 - 0.54) | 2.4E-03 | HMDB12881 | 1 | -0.03 |
| L-NMMA | 0.61 (0.26 - 0.96) | 2.5E-03 | HMDB29416 | 1 | 0.10 |
| N-Methyl-4-pyridone-3-carboxamide | -0.44 (-0.7 - -0.18) | 2.9E-03 | HMDB04194 | 1 | -0.04 |
| Glutamine | 0.51 (0.2 - 0.81) | 3.6E-03 | HMDB00641 | 1 | -0.05 |
| Pyroglutamate | -0.34 (-0.54 - -0.13) | 3.8E-03 | HMDB00267 | 1 | -0.05 |
| Acylcarnitine C14:0 | 0.45 (0.18 - 0.73) | 4.2E-03 | HMDB05066 | 1 | 0.06 |
| Acylcarnitine C8:0 | 0.36 (0.13 - 0.58) | 5.2E-03 | HMDB00791 | 1 | 0.04 |
| Dimethyllysine | 0.32 (0.12 - 0.53) | 5.2E-03 | HMDB13287 | 2 | -0.02 |
| Pantothenate | 0.30 (0.10 - 0.50) | 8.4E-03 | HMDB00210 | 1 | 0.03 |
| 5-Acetylamino-6-amino-3-methyluracil | 0.46 (0.15 - 0.77) | 0.01 | HMDB04400 | 1 | 0.04 |
| Creatinine | 0.33 (0.08 - 0.58) | 0.02 | HMDB00562 | 1 | -0.14 |
| Acylcarnitine C6:0 | 0.35 (0.08 - 0.61) | 0.02 | HMDB00756 | 2 | 0.003 |
| Acylcarnitine C16:0 | 0.37 (0.08 - 0.66) | 0.03 | HMDB00222 | 1 | -0.02 |
| Guanidineacetate | 0.39 (0.08 - 0.71) | 0.03 | HMDB00128 | 1 | -0.04 |
| Methyllysine | 0.17 (0.03 - 0.30) | 0.03 | HMDB02038 | 1 | -0.05 |
| Nicotinamide | -0.45 (-0.83 - -0.08) | 0.03 | HMDB01406 | 1 | -0.05 |
| Taurine | -0.47 (-0.85 - -0.08) | 0.04 | HMDB00251 | 1 | -0.002 |
| 3-Hydroxytrimethyllysine | -0.36 (-0.67 - -0.06) | 0.04 | HMDB01422 | 1 | -0.10 |
| Urobilin | -0.4 (-0.73 - -0.06) | 0.04 | HMDB04160 | 1 | -0.15 |
| 1-Methylhistidine | -0.33 (-0.62 - -0.04) | 0.05 | HMDB00001 | 1 | -0.10 |
| Hypoxanthine | 0.23 (0.01 - 0.46) | 0.07 | HMDB00157 | 1 | -0.03 |
| Acylcarnitine C18:1 | 0.29 (0.01 - 0.57) | 0.07 | NA | 2 | -0.03 |
| Arginine | 0.29 (-0.0 - 0.59) | 0.1 | HMDB00517 | 1 | -0.03 |
| N-Methylproline | 0.28 (-0.0 - 0.58) | 0.1 | HMDB94696 | 1 | 0.07 |
| Tiglylcarnitine | 0.20 (-0.0 - 0.42) | 0.1 | HMDB02366 | 2 | 0.08 |
| Citrulline | 0.25 (-0.0 - 0.51) | 0.1 | HMDB00904 | 1 | 0.02 |
| Acylcarnitine C11:1 | 0.23 (-0.0 - 0.49) | 0.1 | NA | 2 | 0.02 |
| Choline | -0.33 (-0.7 - 0.04) | 0.1 | HMDB00097 | 1 | 0.02 |
| Kynurenate | -0.27 (-0.58 - 0.04) | 0.1 | HMDB00715 | 1 | -0.08 |
| Kynurenine | 0.23 (-0.0 - 0.5) | 0.2 | HMDB00684 | 1 | -0.05 |
| Acylcarnitine C3:0 | -0.21 (-0.47 - 0.05) | 0.2 | HMDB00824 | 1 | -0.08 |
| Urocanate | 0.17 (-0.0 - 0.39) | 0.2 | HMDB00301 | 1 | -0.04 |
| Glycerophosphocholine | -0.24 (-0.54 - 0.07) | 0.2 | HMDB00086 | 1 | -0.01 |
| DMGV | 0.18 (-0.0 - 0.42) | 0.2 | HMDB0240212 | 1 | -0.18 |
| Glutamate | 0.19 (-0.0 - 0.46) | 0.2 | HMDB00148 | 1 | -0.11 |
| Threonine | -0.22 (-0.52 - 0.09) | 0.2 | HMDB00167 | 1 | -0.05 |
| Betaine | 0.17 (-0.1 - 0.45) | 0.4 | HMDB00043 | 1 | 0.02 |
| Asparagine | 0.20 (-0.1 - 0.6) | 0.4 | HMDB00168 | 1 | 0.06 |
| Cotinine | 0.00 (0.0 - 0.0) | 0.5 | HMDB01046 | 1 | -0.11 |
| Methionine | -0.13 (-0.43 - 0.17) | 0.6 | HMDB00696 | 1 | 0.04 |
| Serine | -0.14 (-0.48 - 0.19) | 0.6 | HMDB00187 | 1 | 0.03 |
| 5-Methylthioadenosine | 0.16 (-0.2 - 0.55) | 0.6 | HMDB01173 | 1 | 0.005 |
| Urea | -0.14 (-0.47 - 0.2) | 0.6 | HMDB00294 | 1 | -0.04 |
| Phenylalanine | 0.14 (-0.2 - 0.48) | 0.6 | HMDB00159 | 1 | -0.03 |
| Acylcarnitine C9:0 | 0.10 (-0.1 - 0.37) | 0.7 | HMDB13288 | 2 | 0.09 |
| Leucine | 0.09 (-0.2 - 0.39) | 0.7 | HMDB00687 | 1 | -0.16 |
| Isoleucine | 0.07 (-0.2 - 0.35) | 0.8 | HMDB00172 | 1 | -0.17 |
| Trimethylamine-N-oxide | 0.09 (-0.2 - 0.44) | 0.8 | HMDB00925 | 1 | 0.02 |
| Ornithine | -0.08 (-0.4 - 0.24) | 0.8 | HMDB00214 | 1 | -0.001 |
| Lysine | -0.06 (-0.36 - 0.25) | 0.9 | HMDB00182 | 1 | -0.07 |
| Hydroxycotinine | -0.01 (-0.05 - 0.04) | 0.9 | HMDB01390 | 1 | -0.09 |
| Tryptophan | 0.06 (-0.2 - 0.4) | 0.9 | HMDB00929 | 1 | -0.06 |
| Proline | -0.04 (-0.28 - 0.2) | 0.9 | HMDB00162 | 1 | -0.20 |
| Tyrosine | -0.04 (-0.35 - 0.27) | 1.0 | HMDB00158 | 1 | 0.10 |
| Acylcarnitine C18:0 | 0.04 (-0.2 - 0.33) | 1.0 | HMDB00848 | 1 | -0.001 |
| Acylcarnitine C5:0 | 0.03 (-0.2 - 0.29) | 1.0 | HMDB00688 | 1 | -0.06 |
| Ergothioneine | -0.01 (-0.14 - 0.12) | 1.0 | HMDB03045 | 1 | 0.23 |
| 4-Trimethylammoniobutanoic acid |  |  | HMDB01161 | 1 | -0.15 |
| 25-Hydroxyvitamin D3 | 0.02 (-0.2 - 0.32) | 1.0 | HMDB03550 | 2 | -0.13 |
| Dimethylglycine | 0.01 (-0.1 - 0.18) | 1.0 | HMDB00092 | 1 | -0.10 |
| Methylnicotinamide | -0.02 (-0.42 - 0.38) | 1.0 | HMDB03152 | 1 | -0.01 |
| Methionine-S-oxide | 0.00 (-0.26 - 0.27) | 1.0 | HMDB02005 | 1 | 0.09 |
| Homoarginine | 0.00 (-0.31 - 0.31) | 1.0 | HMDB00670 | 1 | -0.04 |

Measured metabolites and their corresponding change in standard deviation after the intervention. Identify is presented as HMDB-ID and annotation level according to the Metabolomics Standard Initiative Guidelines. Loading: Original loading presented as the loading each metabolite had to the original healthy dietary metabolic signature (HDMS) in the previous publication (1). SD: Standard deviation. Fdr: false discovery rate.

# Supplemental table 2: Changes in original model top associated metabolites

| Positive metabolites | Original model loading | SD change (95 % confidence interval) | Fdr-adjusted p |
| --- | --- | --- | --- |
| Beta-carotene | 0.36 | 0.85 (0.64 - 1.06) | **5,5E-10** |
| Acylcarnitine C4:0-OH | 0.24 | 0.47 (0.27 - 0.68) | **8,5E-05** |
| Ergothioneine | 0.23 | -0.01 (-0.14 - 0.12) | 1 |
| Homostachydrine | 0.21 | 1.06 (0.85 - 1.26) | **1,8E-13** |
| Acylcarnitine C13:0 | 0.21 | 0.81 (0.59 - 1.04) | **9,9E-09** |
| Acetylornithine | 0.20 | 0.68 (0.52 - 0.84) | **2,0E-10** |
| Hippurate | 0.17 | 1.26 (1.07 - 1.46) | **8,8E-17** |
| Proline betaine | 0.15 | 0.48 (0.23 - 0.72) | **6,9E-04** |
| Phenylacetylglutamine | 0.13 | -0.39 (-0.58 - -0.19) | **6,9E-04** |
| Acylcarnitine C13:1 | 0.13 | 0.54 (0.29 - 0.79) | **1,7E-04** |
| Negative Metabolites |  |  |  |
| Proline | -0.20 | -0.04 (-0.28 - 0.2) | 1 |
| DMGV | -0.18 | 0.18 (-0.0 - 0.42) | 0.2 |
| Isoleucine | -0.17 | 0.07 (-0.2 - 0.35) | 0.8 |
| Leucine | -0.16 | 0.09 (-0.2 - 0.39) | 0.7 |
| 4-Trimethylammoniobutanoic acid | -0.15 | 0.03 (-0.2 - 0.35) | 1 |
| Urobilin | -0.15 | -0.4 (-0.73 - -0.06) | **0.04** |
| Creatinine | -0.14 | 0.33 (0.08 - 0.58) | **0.02** |
| 25-Hydroxyvitamin D3 | -0.13 | 0.02 (-0.2 - 0.32) | 1 |
| Acylcarnitine C8:1 | -0.12 | -0.57 (-0.82 - -0.33) | **7.0E-05** |
| Acylcarnitine C10:3 | -0.12 | -0.67 (-0.90 - -0.44) | **1.4E-06** |

Metabolite changes in the 10 metabolites that had the highest positive loadings and 10 highest negative loadings in the original model. SD: Standard deviation. Fdr: false discovery rate.

# Supplementary material references

1. Smith E, Ericson U, Hellstrand S, Orho-Melander M, Nilsson PM, Fernandez C, et al. A healthy dietary metabolic signature is associated with a lower risk for type 2 diabetes and coronary artery disease. BMC Med. 2022;20(1):122.
